# The Usefulness of a Revised Version of the *Material Values Scale—Short Form* in Italian Adolescents: Psychometric Evidence from Two Studies

**DOI:** 10.3390/children11060675

**Published:** 2024-06-02

**Authors:** Carola Beccari, Maria Anna Donati, Giuseppe Iraci Sareri, Caterina Primi

**Affiliations:** 1 Department of Neuroscience, Psychology, Drug, and Child’s Health, Section of Psychology, University of Florence, Via di San Salvi 12, Padiglione 26, 50135 Florence, Italy; mariaanna.donati@unifi.it (M.A.D.); caterina.primi@unifi.it (C.P.); 2 Gruppo Incontro Cooperativa Sociale and CEART (Coordinamento Enti Accreditati Regione Toscana), 51100 Pistoia, Italy; giuseppe.iraci@incontro.coop

**Keywords:** materialism, adolescents, material values scale—short form, risky behaviors, dimensionality, validity

## Abstract

*Background:* Materialism is an attitude that considers material goods to be central in life. Nowadays, adolescents appear to have a high level of materialism, which is related to risky behaviors. Nevertheless, there is a lack of measurement tools with adequate psychometric properties to assess materialism in this age group. For this reason, two studies were conducted to investigate the psychometric properties of the original and short *Material Values Scale* (MVS) in adolescents. *Methods:* In Study 1, participants were randomly split into two subsamples to compare psychometric properties of the original version of MVS with those of the short one. The first subsample consisted of 1054 adolescents (58% male; *M*age = 16.34; *SD* = 1.15), and the second one of 1058 adolescents (57% male; *M*age = 16.26; *SD* = 1.04). In Study 2, the psychometric properties of a revised version of the short MVS (without item 8) were investigated to confirm its adequacy with a new sample composed of 1896 adolescents (60% male; *M*age = 16.40; *SD* = 2.76). *Results:* Results of Study 1 showed that the short version appeared to be a better measuring tool with respect to the long form to investigate materialism in adolescents. Nevertheless, problems with item 8 emerged. Results of Study 2 attested to the adequacy of the psychometric properties of the revised version of the short MVS (by excluding item 8) in this age group, in terms of dimensionality, reliability, and validity. *Conclusions:* Findings show that the revised short version of the MVS could be a valid and reliable tool for measuring the multidimensional construct of materialism in Italian adolescents.

## 1. Introduction

*Materialism* is defined as an individual’s attitude towards giving relevance to the ownership of material goods. Material possessions are regarded as means by which to achieve major life goals or desired end states, particularly those pertaining to individual success and happiness [1]. People with high levels of materialism set their lives around possessions and their acquisition, with the belief that they are essential to improve their image, increase their happiness, achieve a certain social status, and measure their own success in life. A materialistic orientation reflects an individual’s desire to be rich and to own expensive material goods, despite already having money and luxury possessions. 

Although it is a construct of the nineties, materialism turns out to receive particular attention in the XXI century. Several studies have shown that today’s individuals are more materialistic than in the past [2,3], even if materialism seems to have negative effects on well-being [4]. Material orientation is negatively related to well-being, quality of life, and self-esteem [5,6,7], and it is positively related to anxiety and depression [6,8,9]. It is also positively related to work–family conflicts and job dissatisfaction [10,11], and associated with low pro-environmental attitude and behaviors [12]. Materialism is positively related to risky behaviors, such as problem gambling [13], addiction related to new technologies [14,15,16,17], and compulsive buying [18]. It also seems to be involved in cryptocurrencies and online trading, as they are seen as a means to obtain high gains and wealth easily [19,20], but also as a means to gain success and social recognition [21]. 

Considering the negative consequences of materialism in adults, particular attention is paid to materialism in adolescents [22]. Recent studies underline that materialism orientation can manifest early in childhood [23], and even young people seem to ascribe particular importance to material goods [24]. As for adults, materialism in adolescents is related to several negative outcomes, both in personal, social, and scholastic areas [8,25] and to risky behaviors [9,13,26,27,28,29]. Likewise, materialism in adolescents is related to other risky behaviors, such as cigarettes and alcohol [9] and substance use [9].

Considering the relevance that materialism seems to have in different aspects of adolescents’ lives, it is important to pay particular attention to its measurement. Since the late 1980s, several scales have been constructed for measuring materialism, such as *Inglehart’s materialism scale* [30], *Moschis and Churchill’s materialism scale* [31], the *Money Attitude Scale* [32], *Belk’s materialism scale* [33], and the *Consumer Involvement Scale* [34]. Only Goldberg’s *Youth Materialism Scale* [24] investigates this construct in adolescents. However, all these tools present some critical issues regarding the conceptualization of materialism (i.e., a very restrictive definition of materialism) and the adequacy of psychometric properties (i.e., reliability, factor structure) [35]. Moreover, very few studies have used them. To the best of our knowledge, the most used tool to investigate materialism is the *Material Values Scale* (MVS) [1]. The strength of the scale is its multidimensionality. In fact, MVS investigates materialism in terms of three, highly correlated dimensions: *Success*, which refers to an individual’s use of possessions to signal personal success and accomplishment; *Centrality,* which relates to the importance an individual places on acquisition and possessions; and *Happiness*, which corresponds to the general belief that satisfaction with life is brought about by personal possessions. Multidimensional models reflect both the overall situation of the construct and its different aspects through multiple dimensions [36]. Moreover, its psychometric properties have been widely measured and validated in several countries. The original version of the scale is three-dimensional and composed of 18 items with a five-point Likert scale, ranging from 1 (*not true at all*) to 5 (*completely true*). 

The scale performs well in terms of reliability and empirical usefulness [1], but some concerns have been raised regarding the three-factor structure [37]. For these reasons, a short version of MVS [37] was developed. First, three problematic items with low correlation with external criteria and low item–total correlation were deleted (items 6, 7, and 10); then, for each subscale, three items that had the best performance on external, internal, and judgmental criteria were selected, following recommendations provided by Stanton and colleagues [38]. The resulting *MVS—Short Form* [37] was finally composed of nine items that, in line with the original full version [1], have a five-point Likert scale ranging from 1 (*not true at all*) to 5 (*completely true*). Among adults, the short version resulted in maintaining the three-factor structure and showed good psychometric properties both in term of reliability and validity. Moreover, the nine-item scale seemed to display better psychometric properties than the original one in terms of dimensionality and criterion validity [37]. 

Given the increase in materialistic values within society and the risky behaviors that are linked to materialism in adolescence [9,13,26,29], and considering that adolescents are highly exposed to the bombardment of TV advertisements and social media, youth are at risk of sharing their material gain with peers and to being influenced by the material gain of celebrities [39]. Thus, there is a need to have a brief, valid, and reliable tool able to measure the multifaceted construct in this specific age target. The MVS—both in the long and the short version—is commonly used to investigate materialism among adults and young adults, as its psychometric properties have been extensively investigated in these age groups [37,40,41]. However, there is a scan of studies investigating the psychometric properties of the scale in adolescents. Following these premises, we investigated the psychometric properties of these two versions of the scale among Italian adolescents, to validate the better tool to evaluate materialism in this age group. To the best of our knowledge, there are no studies investigating the psychometric properties of these two forms in youth. Only Wang and colleagues [42] analyzed the psychometric properties of a 13-item revised version of MVS [43] in Chinese youth. Although this form was found to be three-dimensional, results concerning dimensionality were not in line with those found by the original authors [1]. 

To this purpose, we conducted two studies. As in the psychometric analysis of an instrument, it is necessary to have at least 10 respondents for each item [44] and at least 200 participants must compose the sample [45]. In both the studies, we recruited large samples. In Study 1, the psychometric properties of the original version of the MVS were compared with those of the short one. We were interested in verifying that the short scale would show better properties in terms of dimensionality, reliability, and validity, with respect to the original version. In Study 2, our aim was to further verify the adequacy of the short version of the MVS, by analyzing its psychometric properties with a new sample of adolescents.

In detail, the goal of Study 1 was to investigate the psychometric properties of the MVS, comparing the original version with the short one composed of nine items. Considering the usefulness of short measurement tools both in research and practice, it is important to investigate if the short version is equivalent or better with respect to the original one and, thus, if it can be profitably used when there is a need to assess the construct briefly. A short form takes up less space on a survey instrument, and thus, it is most useful in cases where researchers want to include additional measures of other constructs with respect to materialism or when materialism is not the primary construct, but it might be a useful variable to be explored. A shorter measure also reduces the likelihood that participants feel fatigued and respond randomly. 

Psychometric properties were analyzed in terms of dimensionality, reliability, and validity. For both the versions, we tested the factor structure through a confirmatory factor analysis, and we investigated validity by considering the correlations with problematic use of videogames, the Internet, and smartphones. Concerning dimensionality, we expected to confirm the three-dimensional structure found by the original authors [1,37]. Concerning the relationships with risky behaviors, we hypothesized high and positive correlations, consistent with the literature [26,27]. We also assessed the reliability of the MVS. Although previous studies have used Cronbach’s alpha as an index of internal consistency for the MVS, this coefficient underestimates the true reliability unless all items have equal covariance with the true score [46,47]. We evaluated internal consistency through McDonald’s omega [48], which does not have this limitation [46,47]. We expected to find good internal consistency values, in line with previous studies, except for the *Centrality* subscale, which was found to have the lowest values [1,37,40,41].

The second study aimed to investigate the psychometric properties of a revised version of the MVS—SF [37], obtained by deleting item 8, focused on *Centrality*, which did not reach good psychometric properties in Study 1. The dimensionality of the new version of MVS—SF was assessed through a confirmatory factor analysis and its reliability was investigated evaluating its internal consistency through McDonald’s omega.

To assess validity, given that materialism turns out to be related to gambling behavior in adolescents [13], we analyzed the correlations with positive expectations of economic gain trough gambling. Additionally, we investigated differences in the revised MVS—SF subscales and total scores by considering adolescents’ gambling status (non-gamblers vs. gamblers), as a criterion. Moreover, considering that behaviors such as cryptocurrencies use and online trading are seen as a mean to obtain high gains and wealth easily [19,20], and are related to gambling in adults [19,49], we also investigated differences in the revised MVS—SF subscale and total scores by considering adolescents’ involvement in cryptocurrencies use (non-cryptocurrencies users vs. cryptocurrencies users) and online trading (non-online traders vs. online traders).

Finally, to keep under control any problems related to the common method variance, linked to the administration of self-report measurement tools, we analyzed the correlations between the subscale scores of the revised MVS—SF with career adaptability. Career adaptability can be defined as a self-managing skill by which individuals interact with the environmental contingencies to cope with changes in career context [50]. As there is no evidence that this construct is related to materialistic orientation, we investigated such relationships with the aim of finding any significant relationship. The absence of correlation would be a proof of validity and, at the same time, a marker against the risk of common method variance [51].

## 2. Study 1

### 2.1. Material and Methods

#### 2.1.1. Participants

We collected data inside the regional gambling prevention program (PRIZE 2.1 [Prevention of gambling risks among adolescents], Resolution of the Tuscany Region n. 1489, 30 November 2020, and Resolution of the Tuscany Region n. 1609, 21 December 2020; Resolution of the Tuscany region entrusted ANCI Toscana (*Association of Tuscan Municipalities*), co-planning with C.E.A.R.T., the implementation of gambling prevention program in school contexts), that was realized during the 2021–2022 school year. The project was aimed at all Tuscany high schools and participation in it was voluntary and free. Thirty-four schools in Tuscany joined the project. The sample was composed of 2112 Italian adolescents (57% male; *M*age = 16.30; *SD* = 1.09) attending public high schools in Tuscany (Italy). Forty-three percent of participants attend a lyceum, forty-one percent a technical school, and sixteen percent a vocational school. The schools involved covered the entire Tuscany territory (North-West, Centre, and South-East) and were representative of the regional and national school population in terms of typology (lyceum, technical and vocational school).

#### 2.1.2. Measures

The MVS [1] has items with a five-point Likert scale ranging from 1 (*not true at all*) to 5 (*completely true*). High scores indicate high levels of materialism. The Italian version (Appendix A) of the MVS was obtained from the English version using the forward translation method. First, two non-professional translators translated the scale independently, and they then assessed equivalence comparing the two versions. Subsequently, a group of five people read and revised the first version, to obtain a final form. The final Italian version of the MVS was translated back to English. We compared the two versions and verified their equivalence.

The second section of the *Videogaming Behavior Scale for Adolescents* [52] was used to measure problematic use of videogames. It is made up of nine items, assessing the nine symptoms of the *Diagnostic and Statistical Manual of Mental Disorders* [53]. An example of an item is “*Did you feel like you could not stop playing videogames?*”. Coefficient omega for the current sample was 0.80.

The *Mobile Phone Problem Use Scale—Short* (MPPUS—S) [54,55] was used to measure adolescents’ problematic use of smartphones. The scale contains ten items on a Likert scale ranging from 1 (*not true at all*) to 5 (*extremely true*), which investigate two correlated factors: *Negative Consequences*, referring to undesirable effects caused by an excessive mobile phone use, and *Dependence*, concerning addiction symptoms. An example of an item is “*It is hard for me to keep my phone off*”. Coefficients omega in the current sample was 0.87.

The short form [56,57] of the *Internet Addiction Test* [58] was used to measure adolescents’ problematic use of the Internet. The scale contains six items on a Likert scale ranging from 1 (*never*) to 5 (*always*), which investigate how often the described behaviors and thoughts are experienced. An example of an item is *“Have your studies been negatively impacted by the amount of time you spend online?”.* Coefficient omega in this sample was 0.84.

#### 2.1.3. Procedure

Students individually completed an anonymous *online* survey. It was administered during school hours under the supervision of trained research assistants. Data collection occurred during the second part of the 2021–2022 school year, from January to March 2022. The survey lasted about 30 min.

#### 2.1.4. Ethics

ANCI TOSCANA (Association of Tuscan Municipalities) declared that the project complied with all requirements. Additionally, each principal and institutional review board of the different high schools reviewed and approved a study protocol created in accordance with the criteria of the Declaration of Helsinki. Participants received an information sheet, and everyone was asked to give written informed assent to participate. Parents received a description of the study and were asked to provide their informant consent (both) to allow the participation of adolescents. Data confidentiality was ensured according to the provisions of General Data Protection Regulation (GDPR 679/2016).

#### 2.1.5. Statistical Analysis

For analysis purposes, participants were randomly split into two subsamples. With the first subsample (*n* = 1054; 58% male; *M*age = 16.34; *SD* = 1.15), we analyzed psychometric properties of the original version of the MVS, while psychometric properties of the short version of the MVS were investigated with the second subsample (*n* = 1058; 57% male; *M*age = 16.26; *SD* = 1.04).

As a preliminary step, normality was assessed. Skewness and kurtosis indices must be between −1 and +1. However, deviation of a few items from normality can be considered negligible [59]. To verify the dimensionality of the scale, a confirmatory factor analysis (CFA) was carried out with AMOS 16.0 [60] using maximum likelihood estimation on the variance–covariance matrix. To analyze the goodness of fit, we considered the comparative fit index (CFI) [61], the Tucker–Lewis index (TLI) [62], and the root mean square error of approximation (RMSEA) [63]. Values above 0.90 indicate an acceptable fit while values above 0.95 indicate an excellent fit of CFI and TLI indices [64]. An acceptable RMSEA value is below 0.08 and good when it is below 0.05 [65]. To estimate reliability, coefficient omega [48] was investigated for all the subscales score of the MVS with JASP 0.18.3 [66]. Validity analyses were performed using bivariate correlations.

### 2.2. Results

#### 2.2.1. Psychometric Properties of the Original MVS

All items presented skewness and kurtosis indices between −1 and +1; thus, normality was verified. However, five items presented low inadequate corrected item–total correlations (Table 1).

**Figure 1:**
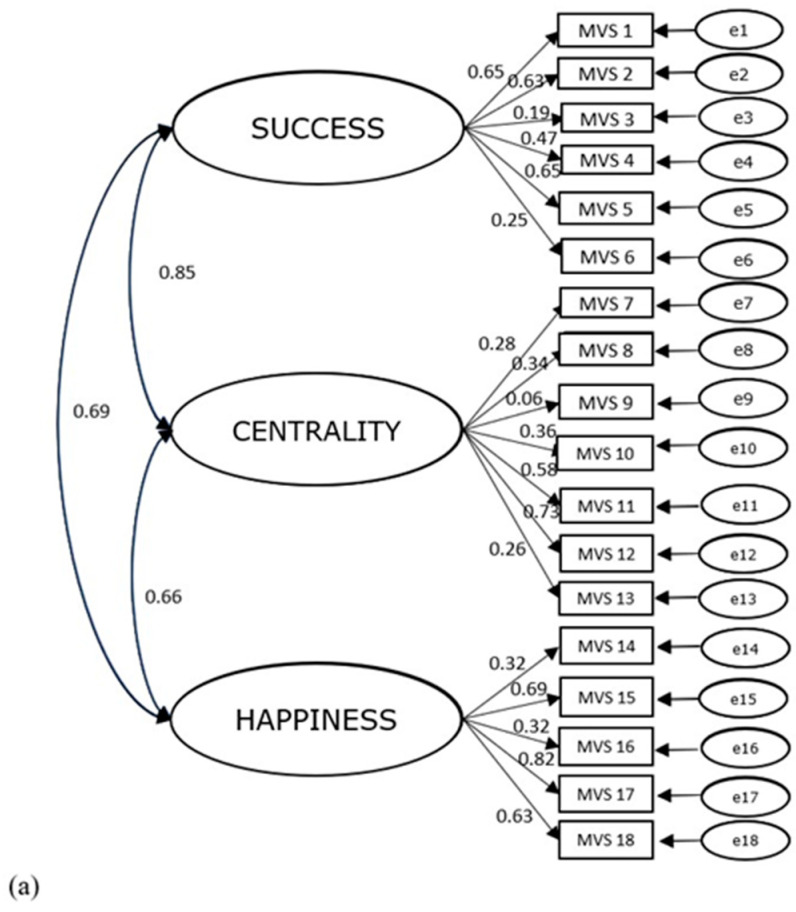

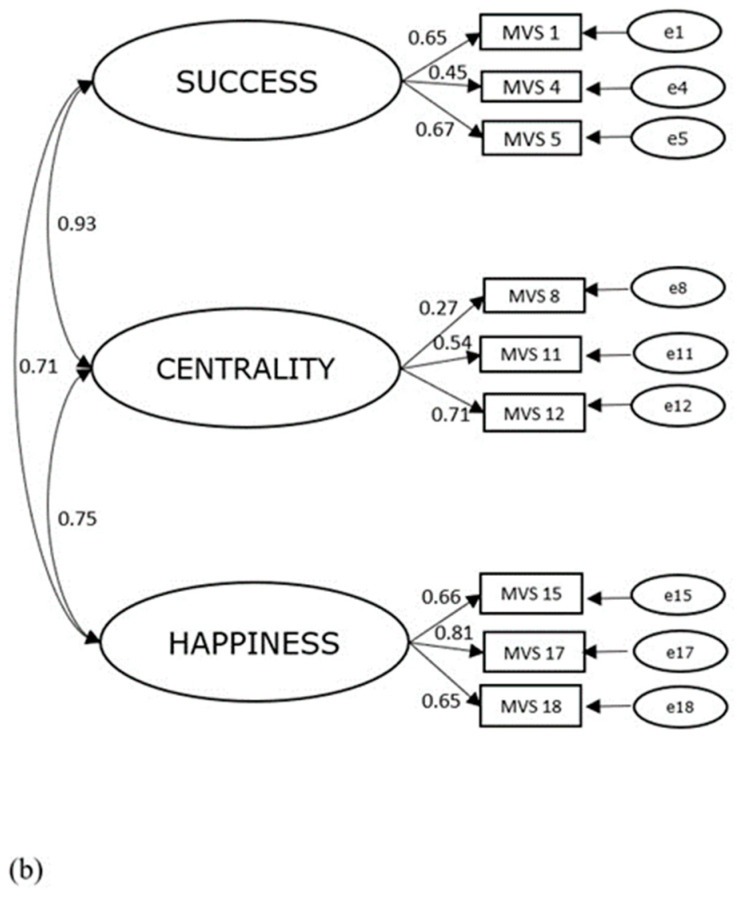
(**a**) Factor structure of the original MVS; (**b**) factor structure of the short version of MVS. *Note:* all factor loadings were significant at the 0.001 level.

Model fit indices showed a poor overall fit: TLI = 0.803, CFI = 0.832, RMSEA = 0.069 (90% CI [0.064, 0.074]), with five standardized factors loadings lower than 0.30 (Figure 1a). Modification indices suggested adding error covariance between items 3 and 6, items 4 and 5, item 7 and 8, and between items 14 and 16. All error covariance occurred between items of the same subscale. Likewise, the modified model showed a poor overall fit: TLI = 0.813, CFI = 0.843, RMSEA = 0.067 (90% CI [0.063, 0.072]), with seven standardized factors loadings lower than 0.30.

Concerning reliability, the omega values were 0.65 for *Success* (95% CI [0.61, 0.68]), 0.57 for *Centrality* (95% CI [0.53, 0.61]), and 0.72 for *Happiness* (95% CI [0.69, 0.75]). The omega value for the total scale was 0.78 (95% CI [0.76, 0.80]). Following the European Federation of Psychological Assessment guidelines [67], the internal consistency values were adequate and good for the *Success* and *Happiness* subscales and for the total score, but inadequate for the *Centrality* subscale.

Concerning validity, positive and significant relationships were found between MVS subscale scores and problematic use of videogames, as well as problematic smartphone use and problematic Internet use (Table 2).

#### 2.2.2. Psychometric Properties of the MVS—Short Form

Normality was confirmed as skewness and kurtosis values were between −1 and +1 for all items. Corrected item–total correlations were acceptable or good with the exception of item 8 (Table 1). Model fit indices confirmed the three-factor structure: TLI = 0.977, CFI = 0.985, RMSEA = 0.036 (90% CI [0.024, 0.048]). Standardized factors loadings ranged from 0.26 to 0.82 and were all significant at the 0.001 level. However, item 8 presented a low inadequate item–total correlation. A high positive linear relationship was found between *Success* and *Centrality*, and moderate positive correlations between *Success* and *Happiness*, and *Centrality* and *Happiness* (Figure 1b).

Concerning reliability, the omega values were 0.63 for *Success* (95% CI [0.59, 0.67]), 0.56 for *Centrality* (95% CI [0.50, 0.064]), and 0.75 for *Happiness* (95% CI [0.72, 0.76]). The omega value for the total scale was 0.80 (95% CI [0.79, 0.82]). The *Centrality* subscale had the lowest internal consistency value.

Concerning validity, significant positive relationships were found between the MVS—SF subscale scores and problematic use of videogames, problematic smartphone use, and problematic Internet use (Table 2).

## 3. Study 2

### 3.1. Material and Methods

#### 3.1.1. Participants

Participants were 1896 Italian adolescents (60% male; *M*age = 16.40; *SD* = 2.76) attending the public high schools in Tuscany (Italy). Half of the participants (about 50%) attended a lyceum, 32% a technical school, and 18% a vocational school. As for Study 1, participants were involved in the second edition of the regional gambling prevention program PRIZE (PRIZE 2.2 [Prevention of gambling risks among adolescents], Resolution of the Tuscany Region n. 1489, 30 November 2020, and Resolution of the Tuscany Region n. 1609, 21 December 2020; Resolution of the Tuscany region entrusted ANCI Toscana (*Association of Tuscan Municipalities*), co-planning with C.E.A.R.T., the implementation of gambling prevention program in school contexts). The schools involved were representative of Tuscany and national high schools. This second edition of the program occurred in the 2022–2023 school year.

#### 3.1.2. Measures

The Italian version (Appendix A) of the revised MVS—SF [37] was used to investigate adolescents’ materialism orientation. In Study 1, the scale is described in detail.

The subscale *Money* of the *Gambling Expectancies Questionnaire* (GEQ) [68], revised and adapted for Italian adolescents [69], was used to investigate adolescents’ positive expectation toward gambling. The subscale consists of three items on a five-point Likert scale, ranging from 1 (*completely disagree*) to 5 (*completely agree*). An example of an item is “*If you were gambling, gambling would make you…getting rich*”. Omega value was 0.85 in this sample.

To assess gambling frequency, the first section of the *Gambling Behavior Scale for Adolescents* (GBS-A) [70] was employed. It includes nine items analyzing gambling frequency in gambling activities such as betting on card games, lotteries, and bets on sporting events, in the last month. Coefficient omega for the current sample was 0.79.

To assess career adaptability, the Italian version [71] of the *Career Adapt-Abilities Scale* (CAAS) [72] was used. It consists of twenty-four items, ranging from 1 (*not strong*) to 5 (*strongest*), which investigate the ways in which adolescents interact with environmental contingencies to cope with present and future changes in career context. The scale measures four related dimensions (*Concern, Control, Curiosity* and *Confidence*) which are combined in a total score. An example of an item is “*Investigating options before making a choice*”. Coefficient omega for the current sample was 0.95 for the total score.

An ad hoc questionnaire was created to analyze adolescents’ knowledge and use of cryptocurrencies and online trading. For cryptocurrencies, questions were *“Do you know cryptocurrencies?”,* and *“Have you ever bought cryptocurrencies?”.* Similarly, for online trading: *“Do you know online trading?”,* and *“Have you ever practiced online trading?”.*

#### 3.1.3. Procedure

Students individually completed an anonymous *online* survey during school hours, under the supervision of trained research assistants, during the first part of the 2022–2023 school year, from November 2022 to January 2023. The survey lasted about 30 min.

#### 3.1.4. Ethics

ANCI TOSCANA (Association of Tuscan Municipalities) declared that the project complied with ethical requirements. As for Study 1, the institutional review boards of each school approved the project. Students were informed that the data obtained would be handled confidentially and anonymously. Parents were informed and asked to provide their informant consent for the participation of their children. Data confidentiality was ensured according to the GDPR 679/2016.

#### 3.1.5. Statistical Analysis

As for Study 1, univariate distributions of the nine MVS—SF items were examined to assess normality. A CFA was carried out with AMOS 16.0 [60] using maximum likelihood estimation, and considering CFI [61], TLI [62], and RMSEA [63] indices to verify the goodness of fit. Concerning reliability, McDonald’s omega was computed for all the subscale scores of the MVS—SF with JASP 0.18.3 [66]. Validity analyses were performed using independent sample *t*-tests.

### 3.2. Results

Normality was confirmed given that skewness and kurtosis indices were between −1 and +1 for all the items. Corrected item–total correlations were good (Table 3). See Appendix A for the correspondence of the number of items of the short version with respect to the original long version.

**Figure 2:**
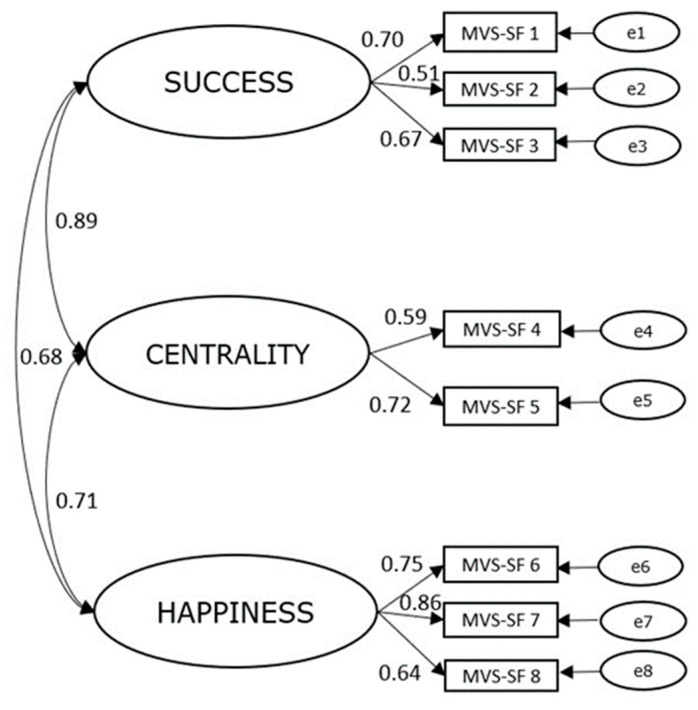
Factor structure of the revised MVS—SF scale. *Note*: all factor loadings were significant at the 0.001 level.

The three-factor structure was confirmed: TLI = 0.969, CFI = 0.981, RMSEA = 0.052 (90% CI [0.042, 0.061]). Standardized factors loadings (range: 0.51–0.86) were significant at the 0.001 level. Positive linear relationships were found between *Success* and *Centrality* (0.89), and moderate correlations were found between *Success* and *Happiness* (0.68), and *Centrality* and *Happiness* (0.71) (Figure 2).

Concerning reliability, the omega values were 0.68 for *Success* (95% CI [0.65, 0.72]), 0.60 for *Centrality* (95% CI [0.56, 0.64]), and 0.79 for *Happiness* (95% CI [0.77, 0.81]). The omega value for the total scale was 0.83 (95% CI [0.82, 0.84]). Omega values were adequate for *Success* and *Centrality*, and good for the *Happiness* and the total score [67]. In line with previous studies, *Centrality* subscale had the lower internal consistency values [37].

**Figure 3:**
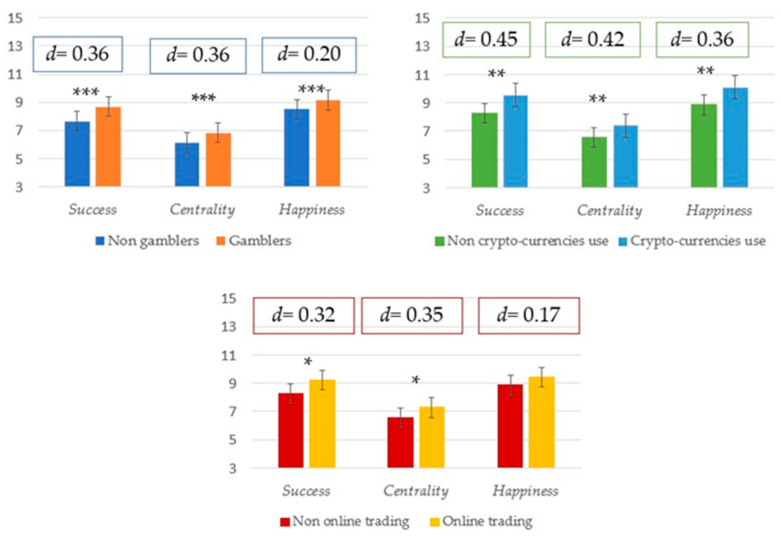
Differences between adolescents who were not involved in gambling behavior and adolescents who were involved in gambling behavior, between adolescents who were not involved in cryptocurrencies use and adolescents who were involved in cryptocurrencies use, and between adolescents who were involved in online trading and adolescents who were not involved in online trading, for the subscales of the revised Material Values Scale—Short Form. *** *p* < 0.001; ** *p* < 0.01; * *p* < 0.05.

Concerning validity, positive and significant relationships were found between the revised MVS—SF subscale scores and positive expectations of economic gain trough gambling. Correlation coefficients were 0.28 for the *Success* subscale, 0.31 for *Centrality*, and 0.24 for *Happiness.* Moreover, as reported in Figure 3, adolescents who gambled (64% males; *M*age = 16.27, *SD* = 1.26) obtained significantly higher scores on the total score and on each subscale compared to those who did not gamble (49% males; *M*age = 16.30, *SD* = 1.24). Cohen’s *d* was −0.36 for the subscale *Success*, −0.36 for *Centrality*, −0.20 for *Happiness,* and −0.035 for the total score. Likewise, adolescents who used cryptocurrencies (95% males; *M*age = 16.45, *SD* = 1.32) demonstrated significantly higher scores on the total score and on each subscale compared to those who did not use them (57% males; *M*age = 16.27, *SD* = 1.25) (Figure 3). Cohen’s *d* was −0.45 for the subscale *Success*, −0.42 for *Centrality*, −0.36 for *Happiness,* and −0.49 for the total score. Additionally, adolescents who practiced online trading (92% males; *M*age = 16.39, *SD* = 1.21) obtained significantly higher scores on the total score and on each subscale, except for *Happiness*, compared to those who did not practice online trading (58% males; *M*age = 16.27, *SD* = 1.26) (Figure 3). Cohen’s *d* was −0.32 for the subscale *Success*, −0.45 for *Centrality*, −0.17 for *Happiness,* and −0.32 for the total score.

Finally, low, and non-significant relationships were found between the revised MVS—SF subscale scores and career adaptability total score. Correlation coefficients were −0.03 for the *Success* subscale, −0.02 for *Centrality*, and −0.08 for *Happiness.*

## 4. Discussion

Study 1 aims to analyze the psychometric properties of the MVS in adolescents, comparing the original version with the short one. Item analyses showed that, for the original version, corrected item–total correlations were low for nine items out of eighteen, while in the short version, item 8 still had a low corrected item–total correlation.

Concerning dimensionality, in line with a previous study [37], model fit indices for the three factors structure were better for the short version, compared to those of the original version. In particular, the long version did not reach the cut off defined for CFI and TLI indices [64]. Moreover, for the original version, standardized factors loadings for five of the eighteen items were lower than 0.30. Instead, the global fit and residual indices of the MVS—SF were found to be excellent in the dimensionality analysis.

As for internal consistency, in line with the original study [37], the MVS—SF subscales maintain an equivalent reliability with respect to the long version, although the number of items was halved compared to the original scale. Moreover, the omega coefficient for the total scale was better for the short version compared to the original. Internal consistency of the *Centrality* subscale was lower with respect to the other subscales, consistent with a previous study [41] that found a low value for the *Centrality* subscale, and in line with studies that evidenced that the internal consistency value for this subscale was lower than for the other subscales, both in the original and in the short version [1,37,41,42]. Concerning validity, in both versions of the MVS, the three subscales had positive relationships with problematic use of videogames, problematic smartphone use, and problematic Internet use, with a greater strength of the correlations for the short form of the MVS with problematic smartphone use. As for the relationship with problematic use of videogames and the Internet, validity evidence was overall equivalent across the two scales. Results confirmed the relationship of materialism with risky behaviors in adolescents [e.g.,13, 26, 27]. It seems to be related to problematic use of smartphones, in line with the fact that a smartphone is a material object that can be considered as indicative of a certain status.

Our findings showed that the original version of the scale presented inadequate psychometric properties in terms of factor structure and reliability. Thus, it appears to be an unreliable tool in measuring materialism in this specific age target. Instead, the short version achieved good psychometric properties both in terms of validity and reliability. Moreover, it maintained its multidimensionality, despite its brevity. Nevertheless, item 8 presented a low inadequate item–total correlation even in the short form, with a factor loading slightly lower than 0.30. For this reason, we conducted a second study with a new sample of youth to investigate the psychometric properties of a revised version of the MVS—SF, where item 8 was deleted. We aimed to validate a short and reliable tool capable of investigating the different components of materialism.

Study 2 aimed to verify the psychometric properties of a revised version of the MVS—SF scale in adolescents. The three-factor structure was confirmed, and a good internal consistency was obtained for the entire scale.

Concerning validity, the revised MVS—SF subscales had positive and adequate in size relationships with positive expectations of economic gain trough gambling, which confirm that materialistic adolescents tend more to use gambling to obtain wealth and success [13]. Moreover, adolescents involved in gambling, cryptocurrency use, and online trading resulted to have a higher materialistic orientation with respect to adolescents not involved in these behaviors, especially those who use cryptocurrencies. Findings confirmed that materialism is related both to gambling-related at-risk factors and to gambling frequency [13]. Moreover, results showed that materialism is related to cryptocurrency use and online trading, providing a contribution to the study of these new types of risky behaviors, which have not yet been investigated in adolescents. Importantly, the revised MVS—SF subscales had no correlation with career adaptability, attesting no problems related to the common method variance linked to self-report measurements [51].

To summarize, our findings confirmed the revised version of MVS—SF is a valid and reliable tool to investigate materialism among adolescents, presenting all the advantages of a short scale. Results also showed that the three dimensions of materialism allow to measure different materialistic behaviors, confirming the usefulness of having maintained the multidimensionality of the scale despite its brevity.

## 5. General Conclusions

The general goal of the present work was to conduct a psychometric analysis of the *Material Values Scale—Short Form* [37], to investigate if it could be an adequate measurement tool to assess materialism in adolescents. Results confirmed the adequacy of its psychometric properties in this age target.

In line with the original authors [1,37], findings of both studies confirm the multidimensional structure with three correlated dimensions: *Success, Centrality*, and *Happiness*. Moreover, results of Study 1 highlighted that the short version of MVS has better psychometric properties than the long one, although some problems with item 8 remained. Results of Study 2 confirmed that a revised version of MVS—SF appears to be more suitable to measure materialism in adolescents.

In line with the literature, our findings confirmed that materialism is related to different types of risky behaviors in adolescents, e.g., [13,26,27]. Despite the notion that materialism is an outdated construct, it constitutes a common basis for different and modern types of risky behaviors in adolescents. Additionally, our work supports the current widespread construct of *homo economicus*, who leverages his competitive positioning and seeks to enhance the value of his capital in all his activities and locations [73].

This work has some limitations. Firstly, the sample consists of Italian high school students, thus caution should be paid in the generalizability of the results. The sample also concerns adolescents who attend school, and therefore may not be generalized to adolescents who do not attend school or have dropped out, who may be more at risk of engaging in risky behaviors. Validity has been also studied by employing self-report instruments, without having objective indicators of adolescents’ purchases and use of money. Moreover, reliability was studied in terms of internal consistency. Although a weakness was found for the *Centrality* subscale, which is in line with a previous study [41], the internal consistency of the total scale resulted to be good.

Future studies should verify the psychometric properties of the scale with students from other countries and with different age groups (e.g., middle-school students or college students). This scale can be profitably used for research and intervention purposes. Concerning research, it could be used to have a measure of materialism in research studies interested in considering materialism as an independent, control, or dependent variable. In terms of independent variables, future studies could investigate the relationships between materialism and other risky behaviors related to the use of money, such as shopping or loot boxes use. Longitudinal studies would be especially useful to investigate whether materialism in adolescents exercises a role in predicting risky behaviors in young adulthood. In detail, given the scarce literature on cryptocurrencies use and online trading in adolescents, future studies should be focused on understanding the relationship between materialism and these new forms of risky behaviors. In parallel, our scale could be employed to assess materialism to test the relationship between materialism and well-being and happiness, as found in adults, e.g., [74,75]. Moreover, studies interested in weighting the specific role of individual and environmental risk and protective factors on pro-environmental behaviors in adolescents, should use this instrument to assess materialism orientation as control variable, given the well-known negative relationship between materialism and behaviors to mitigate climate change’s negative effects, e.g., [76]. Finally, when the research interest focuses on individual values or demographic characteristics that make people more likely to promote a materialistic orientation in adolescents, as in adults [77,78], this scale can be employed to give researchers a relatively easy and fast measure of materialism. As for interventions, future application of this instrument might result in early identification of at-risk students who have a materialistic orientation, and in planning specific forms of intervention. In line with educational programs that aim to develop and cultivate the mental, spiritual, and social dimensions of young people in the aim of becoming part of society, it would be valuable in teaching adolescents to redirect their values on socially accepted norms and rules, especially following the COVID-19 pandemic, in which the usefulness of non-material supports has been tested [79,80,81].

In conclusion, our findings showed that the revised MVS—SF is adequate to measure the multidimensional construct of materialism in adolescents and can be used to investigate the role of materialism in various type of behavioral addictions, especially gambling. Moreover, the strength of this work consists in the replication of the psychometric properties of the scale through Study 2. Thus, the present work demonstrates that the revised MVS—SF provides valuable information about the materialistic orientation of adolescents.

## Figures and Tables

**Table 1:** Means (M), standard deviations (SD), skewness, kurtosis, and item–total correlations of the eighteen items of the MVS and of the nine items of the short form of the MVS.

	*Original MVS*					*Short Version of MVS*				
	*M*	*SD*	Skewness	Kurtosis	*Item–Total* *Correlation*	*M*	*SD*	Skewness	Kurtosis	*Item–Total* *Correlation*
Item 1	3.22	1.22	−0.23	−0.81	0.48	3.17	1.19	−0.27	−0.71	0.43
Item 2	2.94	1.15	0.03	−0.70	0.45					
Item 3	2.95	1.15	−0.12	−0.69	0.19					
Item 4	2.62	1.15	0.19	−0.74	0.34	2.65	1.15	0.12	−0.80	0.39
Item 5	2.79	1.21	0.12	−0.91	0.49	2.77	1.22	0.08	−0.91	0.47
Item 6	3.09	1.10	−0.22	−0.60	0.22					
Item 7	2.76	1.16	0.20	−0.80	0.35					
Item 8	2.74	1.03	0.15	−0.43	0.39	2.72	1.05	0.17	−0.43	0.20
Item 9	2.74	1.14	0.29	−0.72	0.02					
Item 10	2.22	1.21	0.77	−0.32	0.29					
Item 11	3.17	1.19	−0.18	−0.75	0.38	3.17	1.23	−0.15	−0.88	0.34
Item 12	3.20	1.29	−0.17	−1.00	0.32	3.17	1.28	−0.16	−0.95	0.41
Item 13	2.87	1.06	0.06	−0.32	0.22					
Item 14	2.46	1.17	0.45	−0.67	0.38					
Item 15	3.04	1.26	−0.06	−0.99	0.55	3.05	1.25	−0.09	−0.95	0.55
Item 16	3.29	1.16	−0.28	−0.63	0.31					
Item 17	3.26	1.22	−0.26	−0.84	0.58	3.18	1−21	−0.18	−0.80	0.64
Item 18	3.12	1.25	−0.13	−0.98	0.44	3.03	1.26	−0.08	−0.98	0.52

Note: Five-point Likert scale ranging from 1 (*not true at all*) to 5 (*completely true*). MVS: Material Values Scale. On the left side of the table: eighteen items of the original MVS; on the right side of the table: nine items of the original scale that composed the short form of the MVS.

**Table 2:** Bivariate correlations between the Success, Centrality, and Happiness subscale scores and problematic videogame use, problematic Internet use, and problematic smartphone use. Correlations are presented for the MVS and of the short version of the MVS.

	Original MVS			Short Version MVS			Mean (SD)
	Success	Centrality	Happiness	Success	Centrality	Happiness	
VGS-A	0.15 ***	0.17 ***	0.25 ***	0.13 **	0.13 **	0.24 ***	5.30 (3.69)
IAT-SF	0.14 ***	0.12 ***	0.22 ***	0.15 ***	0.17 ***	0.25 ***	13.93 (5.22)
MPPUS	0.24 ***	0.24 ***	0.25 ***	0.25 ***	0.26 ***	0.27 ***	24.07 (8.32)
Mean (SD)	17.56 (4.17)	19.59 (4.28)	15.08 (4.04)	8.61 (2.71)	9.10 (2.52)	9.34 (3.05)	

Note: VGS-A: Video Game Scale for Adolescent; IAT-SF: Internet Addiction Test–Short Form; MPPUS: Mobile Phone Problematic Use Scale, ** *p* < 0.01, *** *p* < 0.001.

**Table 3:** Means (M), standard deviations (SD), skewness, kurtosis, and item–total correlations of the nine items of the MVS—Short Form.

	MVS—Short Form					
	M	SD	Skewness	Kurtosis	Item–Total Correlations	
Item 1	3.06	1.26	−0.09	−0.92	0.49	0.47
Item 2	2.48	1.18	0.30	−0.81	0.26	0.43
Item 3	2.71	1.25	0.23	−0.91	0.45	0.51
Item 5	3.36	1.20	−0.28	−0.79	0.34	0.36
Item 6	3.23	1.28	−0.18	−0.97	0.53	0.44
Item 7	2.99	1.29	−0.04	−1.03	0.56	0.63
Item 8	3.05	1.32	−0.12	−1.08	0.76	0.69
Item 9	2.89	1.31	0.09	−1.11	0.41	0.55

Note: Five-point Likert scale ranging from 1 (not true at all) to 5 (completely true). MVS—SF: Material Values Scale—Short Form.

## Data Availability

The datasets generated during and/or analyzed during the current study are available from the corresponding author on reasonable request. The data are not publicly available due to the fact that the regional gambling prevention program PRIZE 2 [Prevention of gambling risks among adolescents] is still ongoing and it is the property of the project promoter.

## Appendix A

**MVS**	**MVS—SF**	**Italian Version of the Material Values Scale**
Item 1	Item 1	Ammiro le persone che hanno case, auto e vestiti costosi
Item 2		Tra gli scopi più importanti nella vita c’è l’acquistare beni materiali
Item 3		Non considero gli oggetti materiali che le persone hanno come un segno di successo
Item 4	Item 2	Le cose che ho dicono molto sul mio successo nella vita
Item 5	Item 3	Mi piace avere cose che impressionino le persone
Item 6		Non presto molta attenzione agli oggetti che le persone hanno
Item 7		Di solito compro solo cose di cui ho bisogno
Item 8 *	Item 4 *	Per quanto riguarda gli oggetti materiali, provo a mantenere la mia vita semplice
Item 9		Non tutte le cose che ho sono così importanti per me
Item 10		Mi piace spendere denaro in cose che non mi servono
Item 11	Item 5	Comprare cose mi dà tanto piacere
Item 12	Item 6	Mi piace vivere nel lusso
Item 13		Rispetto alla maggior parte delle persone che conosco, dò meno importanza ai beni materiali
Item 14		Ho tutte le cose di cui ho davvero bisogno per godermi la vita
Item 15	Item 7	La mia vita sarebbe migliore se avessi delle cose che non ho
Item 16		Non sarei più felice se avessi cose più belle
Item 17	Item 8	Sarei più felice se potessi permettermi di comprare più cose
Item 18	Item 9	A volte mi dà abbastanza fastidio non potermi permettere di comprare tutte le cose che vorrei

Adapted from ref. [37]. Notes: * item deleted from the revised version of MVS—SF.

**MVS**	**MVS—SF**	**Original Material Values Scale**
Item 1	Item 1	I admire people who own expensive homes, cars, and clothes.
Item 2		Some of the most important achievements in life include acquiring material possessions.
Item 3		I don’t place much emphasis on the amount of material objects people own as a sign of success.
Item 4	Item 2	The things I own say a lot about how well I’m doing in life.
Item 5	Item 3	I like to own things that impress people.
Item 6		I don’t pay much attention to the material objects other people own. *
Item 7		I usually buy only the things I need.
Item 8 *	Item 4 *	I try to keep my life simple, as far as possessions are concerned.
Item 9		The things I own aren’t all that important to me.
Item 10		I enjoy spending money on things that aren’t practical.
Item 11	Item 5	Buying things gives me a lot of pleasure.
Item 12	Item 6	I like a lot of luxury in my life.
Item 13		I put less emphasis on material things than most people I know.
Item 14		I have all the things I really need to enjoy life.
Item 15	Item 7	My life would be better if I owned certain things I don’t have.
Item 16		I wouldn’t be any happier if I owned nicer things.
Item 17	Item 8	I’d be happier if I could afford to buy more things.
Item 18	Item 9	I admire people who own expensive homes, cars, and clothes.

Notes: * item deleted from the revised version of MVS—SF.
